# The potential for improving cardio-renal outcomes by sodium-glucose co-transporter-2 inhibition in people with chronic kidney disease: a rationale for the EMPA-KIDNEY study

**DOI:** 10.1093/ckj/sfz009

**Published:** 2019-02-11

**Authors:** William G Herrington, David Preiss, Richard Haynes, Maximilian von Eynatten, Natalie Staplin, Sibylle J Hauske, Jyothis T George, Jennifer B Green, Martin J Landray, Colin Baigent, Christoph Wanner

*Clinical Kidney Journal*, Volume 11, Issue 6, 1 December 2018, Pages 749–761, https://doi.org/10.1093/ckj/sfy090

In the footnote of Figure 5 “Empagliflozin 0/4687 vs placebo 1/2333 participant reported Fournier's gangrene (perineal necrotizing fasciitis)” has been replaced with “Empagliflozin 0/4687 vs placebo 0/2333 participants reported Fournier’s gangrene (perineal necrotizing fasciitis)”.

